# Multipronged strategy for protection and motivation of healthcare workers during the COVID-19 pandemic: a real-life study

**DOI:** 10.1017/ash.2023.459

**Published:** 2024-07-25

**Authors:** Madhumita Premkumar, Usha Dutta, Anchal Sandhu, Harman Kaur, Mini P Singh, Kapil Goyal, Rashmi Ranjan Guru, PVM Lakshmi, Madhu Gupta, Manisha Biswal, Arnab Ghosh, Anurag Sachan, Shikha Guleria, Swapanjeet Sahoo, Sandeep Grover, Tulika Gupta, Vipin Koushal, Mahesh Devnani, Shweta Talati, Ritin Mohindra, Vikas Suri, RK Ratho, Ashish Bhalla, Sanjay Jain, Pankaj Arora, Navin Pandey, Ashok Kumar, Arun K. Aggarwal, Arunaloke Chakrabarti, Goverdhan Dutt Puri, Jagat Ram, SS Pandav, Rakesh Sehgal, Pankaj Malhotra, Narayana Yaddanapuddi, Surjeet Singh, PGIMER COVID-19 Healthcare Worker Welfare Taskforce

**Affiliations:** 1 Department of Hepatology, Post Graduate Institute of Medical Education and Research (PGIMER), Chandigarh, India; 2 Department of Gastroenterology, Post Graduate Institute of Medical Education and Research (PGIMER), Chandigarh, India; 3 Department of Virology, Post Graduate Institute of Medical Education and Research (PGIMER), Chandigarh, India; 4 Department of Hospital Administration, Post Graduate Institute of Medical Education and Research (PGIMER), Chandigarh, India; 5 Department of Community Medicine and School of Public Health, Post Graduate Institute of Medical Education and Research (PGIMER), Chandigarh, India; 6 Department of Microbiology, Post Graduate Institute of Medical Education and Research (PGIMER), Chandigarh, India; 7 Department of Psychiatry, Post Graduate Institute of Medical Education and Research (PGIMER), Chandigarh, India; 8 Department of Anatomy Post Graduate Institute of Medical Education and Research (PGIMER), Chandigarh, India; 9 Department of Internal Medicine, Post Graduate Institute of Medical Education and Research (PGIMER), Chandigarh, India; 10 Department of Anesthesia, Post Graduate Institute of Medical Education and Research (PGIMER), Chandigarh, India; 11 Department of Ophthalmology, Post Graduate Institute of Medical Education and Research (PGIMER), Chandigarh, India; 12 Department of Parasitology, Post Graduate Institute of Medical Education and Research (PGIMER), Chandigarh, India; 13 Department of Hematology, Post Graduate Institute of Medical Education and Research (PGIMER), Chandigarh, India; 14 Department of Pediatrics, Post Graduate Institute of Medical Education and Research (PGIMER), Chandigarh, India

## Abstract

**Objective:**

We aimed to assess risk of COVID-19 infection & seroprotection status in healthcare workers (HCWs) in both hospital and community settings following an intensive vaccination drive in India.

**Setting:**

Tertiary Care Hospital

**Methods:**

We surveyed COVID-19 exposure risk, personal protective equipment (PPE) compliance, vaccination status, mental health & COVID-19 infection rate across different HCW cadres. Elecsys® test for COVID-19 spike (Anti-SARS-CoV-2S; ACOVs) and nucleocapsid (Anti-SARS-CoV-2; ACOV) responses following vaccination and/or COVID-19 infection were measured in a stratified sample of 386 HCW.

**Results:**

We enrolled 945 HCWs (60.6% male, age 35.9 ± 9.8 years, 352 nurses, 211 doctors, 248 paramedics & 134 support staff). Hospital PPE compliance was 90.8%. Vaccination coverage was 891/945 (94.3%). ACOVs neutralizing antibody was reactive in 381/386 (98.7%). ACOVs titer (U/ml) was higher in the post-COVID-19 infection group (N =269; 242.1 ± 35.7 U/ml) than in the post-vaccine or never infected subgroup (N = 115, 204.1 ± 81.3 U/ml). RT PCR + COVID-19 infections were documented in 224/945 (23.7%) and 6 HCWs had disease of moderate severity, with no deaths. However, 232/386 (60.1%) of HCWs tested positive for nucleocapsid ACOV antibody, suggesting undocumented or subclinical COVID-19 infection. On multivariate logistic regression, only female gender [aOR 1.79, 95% CI 1.07–3.0, *P* = .025] and COVID-19 family contact [aOR 5.1, 95% CI 3.84–9.5, *P* < .001] were predictors of risk of developing COVID-19 infection, independent of association with patient-related exposure.

**Conclusion:**

Our HCWs were PPE compliant and vaccine motivated, with immunization coverage of 94.3% and seroprotection rate of 98.7%. There was no relationship between HCW COVID-19 infection to exposure characteristics in the hospital. Vaccination reduced disease severity and prevented death in HCW.

## Introduction

Health care personnel (HCWs) remain at high risk of being infected by SARS-CoV-2 despite the availability of efficacious vaccines.^1^ In India, from 3 January 2020 to 23 June 2022, there have been 43,362,294 confirmed cases of COVID-19 with 524,954 deaths, reported to the World Health Organization (WHO). India rolled out the COVID-19 vaccine program to all HCWs as a priority from 16th January 2021 onward. As of 15 June 2022, a total of 1,956,513,732 vaccine doses have been administered.^2^ We have previously described our holistic HCW-centric COVID-19 policy to ensure logistic support, and appropriate personal protective equipment (PPE) with training to ensure a rolling cohort of motivated HCWs at our nodal COVID-19 facility. To establish a long-term HCW-centric policy, we need to factor in the likelihood of a long-duration COVID-19 pandemic with multiple waves and waning HCW immunity.^3^ Fear of COVID-19 and death had demotivated many HCW which was ameliorated by our multipronged approach to protect HCW.^4^ There is new data regarding sensitivity and specificity of antibody assays used for the purpose of seroprevalence, and most reports have used the anti-spike or nucleocapsid antibody assays.^5–7^ Therefore, we designed an HCW-centric COVID-19 care model adopted by our center to protect HCW from the rigors of extended COVID-19 duties. We engaged actively with HCW, mitigated their fears, and prepared them for these duties. In this prospective cohort study from a single large center in a developing country, we aimed to assess the vaccine coverage, seroprotection status, PPE compliance, and risk of developing COVID-19 in our cohort of HCW across healthcare cadres, area of deployment, and levels of exposure in a resource-limited setting. We assessed the real-life protection measures adopted by our center to protect HCW in a resource-limited setting.

## Methods

### Study design

**Figure 1.**
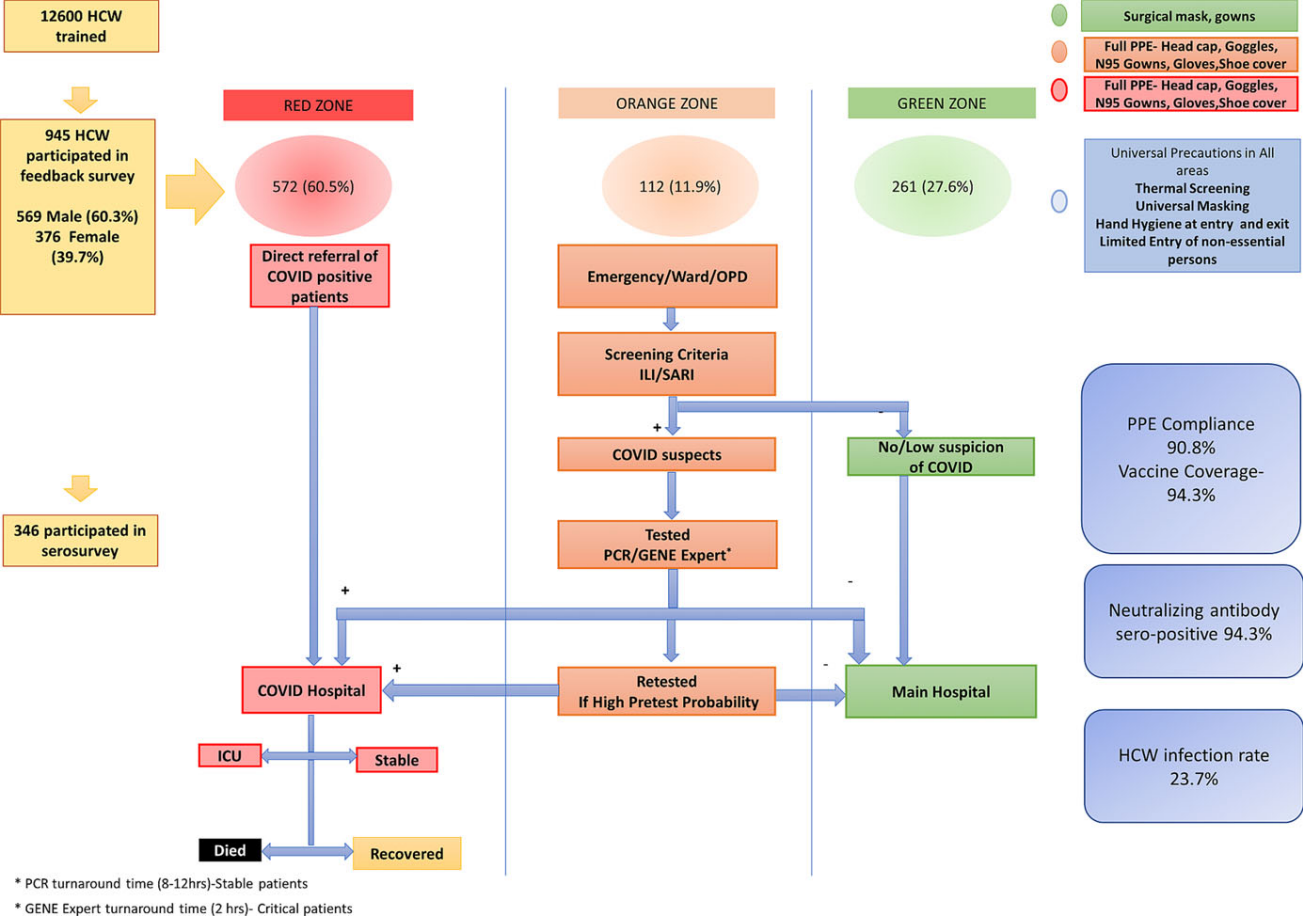
Creation of Infectious Disease Control Zones in the hospital and designation of levels of Personal Protective Equipment (PPE) based on HCW deployment. (Adapted From Dutta U, *et al* BMJ Open 2021;11:e043837. doi: 10.1136/bmjopen-2020-043837).

The Postgraduate Institute of Medical Education and Research (PGIMER), Chandigarh, India, was designated as a COVID-19 treatment center on 21 March 2020. This is a tertiary care university hospital with 2800 beds catering to multidisciplinary referred cases from Northern India, specifically serving the states of Punjab, Haryana, Himachal Pradesh, and western Uttar Pradesh. The COVID-19 HCW welfare team was created to formulate HCW-centric policies and ensure logistic support for our frontline team.^3,8^ The COVID-19 screening was performed in the orange zone including the emergency room, outpatient unit, and severe acute respiratory infection (SARI) ward, which had allocated PPE including N95 masks, face shields, and surgical gowns. Patients who tested positive were managed in the exclusive COVID-19 facility (red zone) with 323 beds, including 74 intensive care and 99 high-dependency beds. The red and orange zones had strict full PPE gear allocation. The rest of the hospital was designated as a green zone wherein universal masking and standard protective measures were adopted.^3^ (Figure 1).

All 12,600 HCWs in the PGIMER were trained based on deployment zone and followed PPE instructions specific to their area of work. (Supplementary information). The study was approved by the Institutional Ethics Committee (NK/7472/Study/302 dated 10/6/21). The inclusion criteria were all HCWs who worked in the COVID-19 facility and at other sites in the hospital. Exclusion criteria were HCWs who were in quarantine or had RT-PCR-confirmed SARS-CoV2 infection at the time of serosurvey. All HCWs provided written informed consent before inclusion and serum samples were collected and stored at minus 80 degrees Celsius. Between August to September 2021, a stratified sample based on age, gender, and HCW cadre to provide a cross-sectional assessment of seroprotection. The number of study participants was determined based on voluntary participation, higher presumed hospital-related COVID-19 exposure in red and orange zones, and feasibility of immunological analyses A cloud-based case record form was used to collect relevant clinical, demographic data, exposure with suspected or confirmed COVID-19 patients or in the community, PPE compliance in the hospital and in the community, comorbid ailments, and use of HCW support facilities extended to them by the hospital like transport, accommodation, rest periods, etc. Details of vaccination, dose interval, and number of doses were noted.

### Definitions

The WHO defines healthcare workers (HCW) as “all people engaged in actions whose primary intent is to enhance health.”^9^ HCW were defined as “paid and unpaid persons serving in health care settings who have the potential for direct or indirect exposure to patients or infectious materials.”^10^

COVID-19 Infection: All HCWs who ever tested positive on COVID-19 RT-PCR testing were reported as *RT-PCR + confirmed COVID-19 infections*.^11^ Persons who tested anti-SARS-CoV-2 nucleocapsid antibody reactive, without a documented positive RT-PCR test, were taken as *presumed COVID-19 infections* who remained untested, because of mild or minimal symptoms. Severity of COVID-19 disease was reported as per ICMR guidelines.^12^

### Neutralization assays

We selected a subset of study volunteers for seroprotection analysis based on matching for sex and age to represent those who were more likely to have higher in-hospital exposure to COVID-19 and also determine gender-based differences in seroprotection status, and also determine variations based on cadre i.e. surgeons vs physicians, nursing vs doctor vs paramedical staff, etc. We assessed the presence of SARS-CoV-2-specific antibodies using a microarray-based immunoassay including spike protein (full spike and nucleocapsid protein as antigens to discriminate between vaccine-induced antibody response and convalescent SARS-CoV-2 infection.^13^ SARS-CoV-2 specific neutralizing antibody responses were characterized by antibodies including IgG against spike protein using Elecsys®Anti-SARS-CoV-2s (ACoVs) manufactured by Roche Diagnostics International Ltd CH, Rotkreuz, Switzerland. Result of SARs-CoV-2 spike assay titer ≥ 250 IU/mL is interpreted as reactive. The nucleocapsid assay was measured by Elecsys®Anti-SARS-CoV-2 N (ACoV) test, with titer ≥ 50 IU/mL reported as reactive. This test was positive in those with clinical or subclinical COVID-19 infection. The tests were done on the Cobas e 411 analyzer.^14,15^

## Statistical analysis

Descriptive statistics were expressed as mean with standard deviation for parametric data and median with interquartile range for non-parametric continuous data. Categorical data were reported as numbers (n, %). We used Student’s *t*-test to compare continuous data between the two groups. We used Pearson’s Chi-square test or Fisher’s exact test (as appropriate) to compare categorical data among the two groups or ANOVA test for comparison between multiple groups. Binary logistic regression was performed for predictors of developing COVID-19 infection, with multivariate analysis for adjusted risk. The main outcomes are reported as estimated effect sizes along with precision (95% confidence intervals [CIs]). Statistical significance was set at *P* < .05. We conducted the analysis using SPSS ver. 23.0 (IBM Corp., Armonk, NY, USA).

## Results

**Figure 2.**
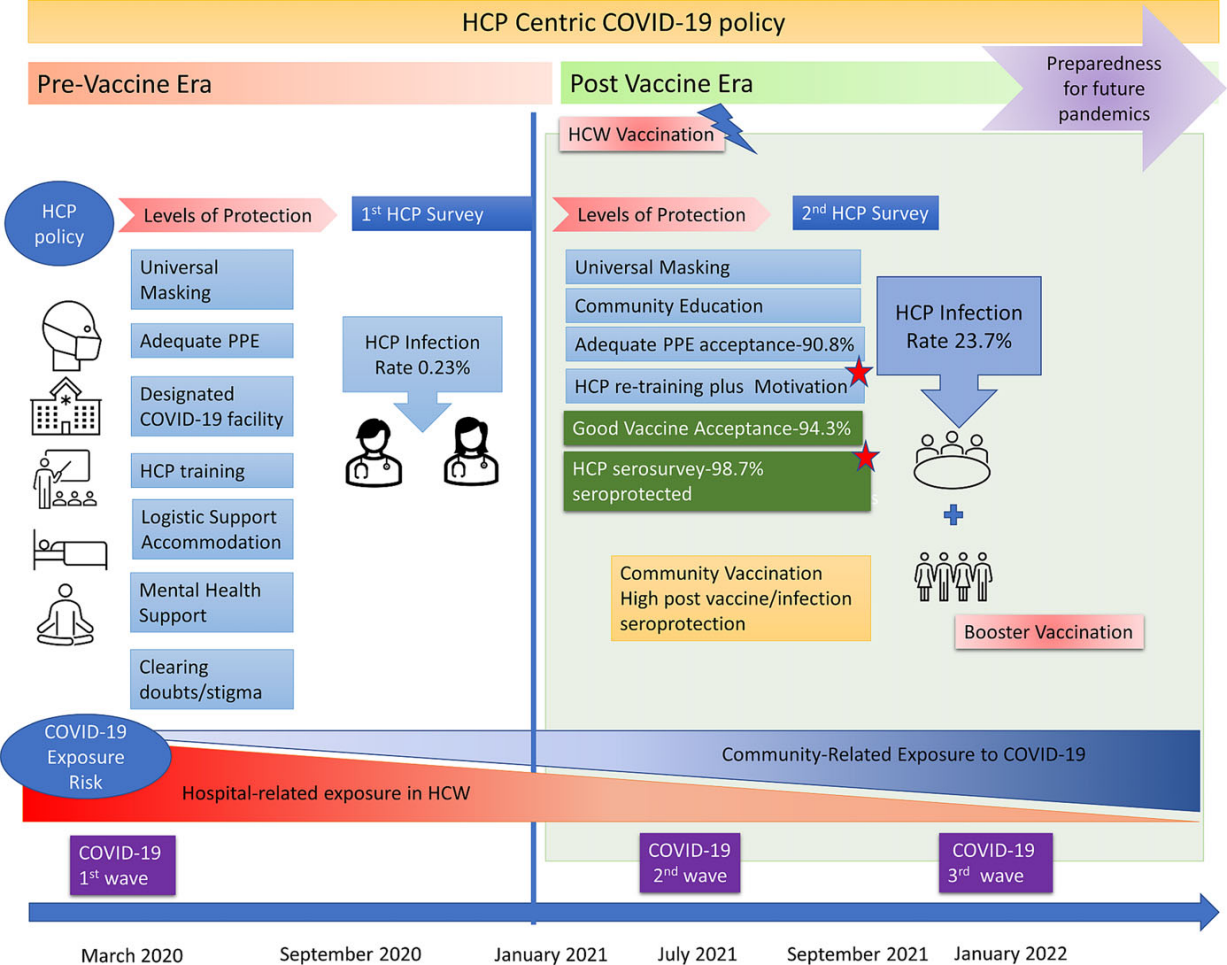
Recommendations from an HCW-centric approach to future public health challenges to protect and motivate HCW, and ensure cost effective, sustainable frontline workforce, while maintaining routine health care services without disruption.

We enrolled 945 HCW (569/945, 60.2% male, mean age 34.0 ± 8.5 yr) who had served in the COVID-19 facility, intermediate risk COVID-19 areas (Orange Zone), and low-risk areas (Green Zone) including 352 nurses, 211 doctors, 248 paramedics, 103 support & administration and 31 research staff. The HCWs were posted in the red zone(572/945, 60.5%), intermediate risk or orange zone (112/945, 11.9%), and green zone (261/945, 27.6%). Ninety-five (95, 10.1%) reported a comorbid illness including hypertension (23, 24.2%), hypothyroidism (23, 24.2%) diabetes (14, 14.7%), asthma (7, 7.3%), allergies (4, 4.2%), nonalcoholic fatty liver disease (4, 4.2%), and rheumatological disorders. (9, 9.4%) Figure 2 shows the deployment of the HCW and exposure levels as per hospital zones.

The total number of COVID-19 patients managed at the COVID-19 facility from March 30, 2020, till September 30, 2021, included 5488 patients (61.8% male) of whom 1159 (21.1%) were managed in the COVID-19 ICU and 4329 (78.8%) were admitted to the COVID facility wards. There were 1002 deaths (18.2%) as mainly sick patients with comorbidities &organ failures were referred to our hospital.

Table 1 shows the classification of HCWs as per their infection and vaccination status to assess the degree of protection afforded by our HCW-centric care policy and the vaccination facility provided to our HCW. We serosampled 386/945 (40.8%) of our HCW.

**Table 1.** Baseline characteristics of the subcategories of serosurveyed HCW (N = 386) based on infection/vaccination status to assess deployment, PPE usage, and community risk

	Total	Not vaccinated		Vaccinated		
Parameter	All HCW	No COVID-19 infection	Presumed COVID-19 infection	No COVID-19 infection	Presumed COVID-19 infection^a^	
HCW number (%)	n = 386 (100)	n = 2 (0.5)	n = 3 (0.8)	n = 115 (29.8)	n = 266 (68.9)	*P* value
Male	234 (60.6)	1 (50.0)	1 (33.3)	69 (60.0)	163 (61.3)	.778
Age (yr)	35.9 ± 9.8	36.5 ± 3.5	46.0 ± 14.1	36.6 ± 9.8	35.7 ± 9.7	.348
Age Category						
<35 yr	225 (58.3)	1 (50.0)	1 (33.3)	63 (54.8)	160 (60.2)	.286
35–55 yr	143 (37.0)	1 (50.0)	1 (33.3)	45 (39.1)	96 (36.1)	
>55 yr	18 (4.7)	0	1 (33.3)	7 (6.1)	10 (3.8)	
Comorbidities	43 (11.1)	0	0	14 (12.1)	29 (10.9)	.078
	Diabetes,^8^ HT,^14^ Obesity,^6^ Hypothyroidism,^5^ Rheumatoid arthritis^2^ Asthma,^2^ Allergies,^2^ Peripheral neuropathy^1^			Diabetes,^5^ HT,^6^ Obesity,^1^ Rheumatoid arthritis^1^	Diabetes,^8^ HT,^8^ Obesity,^5^ Hypothyroid,^5^ Rheumatoid arthritis,^1^ Asthma,^2^ Allergies,^2^ Peripheral neuropathy^1^	
Hospital zone of posting						.742
Red (COVID-19 facility)	137 (35.5)	1 (50.0)	0	41 (35.7)	95 (35.7)	
Orange (emergency or SARI Ward)	34 (10.3)	0	0	10 (8.7)	30 (11.3)	
Green (remainder of the hospital)	209 (54.1)	1 (50.0)	3 (100)	64 (55.7)	141 (53.0)	
PPE in hospital						.680
Surgical mask	100 (25.9)	0	1 (33.3)	38 (33.0)	61 (22.9)	
N95 only	161 (41.7)	1 (50.0)	2 (66.7)	44 (38.3)	114 (42.8)	
Double mask (N95 plus surgical)	125(32.3)	1 (50.0)	0	33 (28.7)	91 (34.2)	
PPE in community wear						.301
Cloth mask	87 (5.74)	1 (50.0)	1 (33.3)	21 (18.2)	64 (24.06)	
Surgical mask	143 (37.04)	1 (50.0)	1 (33.3)	41 (35.6)	100 (37.5)	
N95 only	156 (40.4)	0	1 (33.3)	53 (46.1)	102 (38.3)	
Family member tested positive (Community exposure)	81 (22.6)	0	2 (66.7)	17 (14.8)	62 (26.1)	.023

Abbreviations: HCW, healthcare worker; PPE, personal protective equipment; wk, week.

a Presumed COVID-19 infection includes RT-PCR positive COVID-19 infected HCW (N = 224) and anti-ACoV nucleocapsid positive persons (N = 42) in those who are vaccinated.

The 386 HCWs could be classified into 4 categories, 115 (29.8%) had received the COVID-19 vaccine and never developed overt RT PCR + or asymptomatic (anti-ACoV nucleocapsid positive) COVID-19 infection. Again 266 (68.9%) had received the vaccine and had also tested positive for COVID-19 infection, of whom 224 were RT PCR positive infections and 42 were anti-ACoV nucleocapsid positive subclinical infections. The last two categories were those who were infected and waiting to be vaccinated [3 (0.8%)] and those who never were vaccinated or infected with COVID-19 [2 (0.5%)]. Supplementary Table 1 shows the exposure risk and tours of duty in the COVID-19 facility.

Of all the 945 HCWs, 572 (60.5%) reported an exposure to a COVID-19-positive individual either in the hospital or in the community during the last 1 year with equal self-perceived risk exposure in male and female HCWs. Importantly 300 (31.7%) could not recall if there was any breach in PPE and could not identify an exposure. The long exposure or repeated tours of duty were noted in specialized HCWs like anesthesia, dialysis, and endoscopy technicians or ICU nursing personnel. In total 208 (22.0%) of our cohort had completed >2 months of cumulative duty in COVID-19 areas. The majority (52.5%) reported a daily exposure of 6-8 hours only, as we restricted workers to 6-hour shifts in PPE, to allow time for physical and mental recovery. Many HCWs indicated a preference for dual masks with N95 + surgical (449/945, 47.5%) or N95 alone (340/945, 36.0%) in the COVID red and orange zones. Compliance with full PPE was 90.8% in the COVID-19 utility.

### Vaccination status and seroprotection

Vaccination coverage was 891/945 (94.3%), of whom 723/945 (76.5%) received 2 doses, 168/945 (17.8%) had received 1 dose, and 54/945 (5.7%) deferred vaccination due to recent COVID-19 in compliance with guidelines. HCW mainly received ChAdOx1 875/891, 98.2%) vaccine, as it was provided at our site. Post-COVID vaccine mild adverse effects were reported in 281 (31.5%) including fever (74, 26.3%), myalgia (66, 23.4%), sore throat (25, 8.89%), injection site complaints (50, 17.7%), headache (25, 8.8%), diarrhea (3, 1.06%) and 38 (13.5%) had malaise. No major adverse events requiring hospitalization were noted. The vaccine dose interval was mostly 4–8 weeks 131 (33.9%), and 274 (28.9%) had a gap of >8 weeks.

The **Anti-SARS-CoV-2** (ACoV) nucleocapsid test was reactive in 232/386 (60.1%) persons of the 386 HCW who underwent the serological testing, including 366/381 (95.1%) of the persons who were vaccinated and 3/5 (60%) of the persons who were not vaccinated yet suggesting significant subclinical or undiagnosed COVID-19 infection in our HCW.

The **Anti-SARS-CoV-2s** (ACoVs) test was reactive in 366/386 (95.1%) subjects who underwent the serosurvey, including 381/386 (98.7%) vaccinated individuals and 5/386 (1.2%) unvaccinated individuals. Age group and gender did not predict attainment of protective titer. (Table 2). Overall maximum immunity was attained by those who developed both COVID-19 infection and were vaccinated. The protective antibody was 243.1 ± 33.7 U/ml in those with both vaccination and prior COVID-19 infection, and 204.1 ± 81.3 U/ml in those who were vaccinated and never had clinical or subclinical infection.

**Table 2.** Vaccination details of the 386 HCW in the serosurvey and results of Anti ACoV antibody and Anti ACoVs antibody tests based on vaccination status

		Not vaccinated		Vaccinated		
Parameter	Total	No COVID-19 infection	Presumed COVID-19 infection	No COVID-19 infection	Presumed COVID-19 infection^a^	
HCW number (%)	N = 386	N = 2 (0.2)	N = 3 (0.3)	N = 115 (12.2)	N = 266 (68.9)	*P* value
Type of vaccine						
* Covaxin* ^*TM ®*^	6 (1.6)	0	0	3 (2.6)	3 (1.1)	
* Covishield* ^*TM ®*^	374 (96.9)	0	0	112 (97.4)	262 (98.5)	
* Sputnik V* ^*TM ®*^	1 (0.3)	0	0	0	1 (0.4)	
Number of Doses						
* Single*	43 (11.1)	0	0	11 (9.6)	32 (12)	.000
* Both doses*	338 (87.6)	0	0	104 (90.4)	234 (88.0)	
* None*	5 (1.5)	2 (100)	3 (100)	0	0	
Dose interval						
Not vaccinated	5 (1.5)	2 (100)	3 (100)	0	0	
<4 wk	3 (0.8)	0	0	2 (1.7)	1 (0.4)	
4 wk	96 (24.8)	0	0	34 (29.5)	62 (16.06)	
4–6 wk	86 (22.3)	0	0	23 (20)	63 (23.7)	
6–8 wk	45 (11.7)	0	0	15 (13)	30 (11.3)	
>8 wk	151 (39.1)	0	0	41 (35.7)	110 (41.4)	
ACoV anti-nucleocapsid						
ACoV titer U/mL	193.1 (140.0–318)	0	81.8(95.0–318.0)	44.1(23.7–85.9)	269.1 (145–345)	.000
ACov anti-spike						
Reactive N (%)	366(94.8)	0	3 (100)	105 (91.3)	258 (97)	.002
ACoV-2 Spike antibody titer U/mL	230.9 ± 56.5	–	250.0 ± 0	204.1 ± 81.3	243.1 ± 33.7	.000

Abbreviations: ACoV, Anti-SARS-CoV-2 nucleocapsid test; ACoVs, Anti-SARS-CoV-2s spike test.

a Presumed COVID-19 infection includes RT-PCR positive COVID-19 infected HCW (N = 224) and anti-ACoV nucleocapsid positive persons (N = 42) in those who are vaccinated.

### COVID-19 infections and predictors of infection

Overall, COVID-19 infections were noted in 224/945(23.7%) of this cohort of 945 HCWs during the first and second wave. The RT-PCR-positive COVID-19 infection rate was 0.23% in the 1^st^ wave, which increased to 23.7% during the second wave, possibly due to increased case burden and infectivity of the delta variant. PPE compliance was 90.8% in the COVID-19 facility but only 60.5% in the community. Most HCWs reported an exposure in the family or the community or with co-workers in lounge areas or cafeteria. Of 945 HCWs, 193/945 (20.4%) reported a first-degree relative testing positive during the last 1 year. There was no difference in the infection rates in surgical and medical units or in clinical or paraclinical units. On logistic regression, we tested predictors of COVID-19 infection in our cohort based on age, gender, cadre, site of deployment, duration of exposure, cumulative duty, vaccination status, dose interval, neutralizing antibody level, etc. Presence of a COVID-19-positive family contact was the strongest predictor of infection (adjusted Odds Ratio (aOR) 5.15, 95% CI 3.84–9.5, *P* = .000). This suggested community origin of transmission, rather than a hospital source. Women had a higher risk of developing infection (aOR 1.79, 95% CI 1.07–3.0, *P* = .025). (Table 3)

**Table 3.** Results of binary logistic regression for predictors of COVID-19 infection in HCW

	Univariate			Multivariate		
Variable	OR	95% CI	*P* value	adjusted OR	95% CI	*P* value
Female gender	1.77	1.31–2.39	.000	1.79	1.07–3.0	.025
Family members testing positive	4.62	3.29–6.47	.000	5.15	3.84–11.5	.000

Abbreviations: aOR. Adjusted Odds Ratio; HCW, health care workers.

### Mental health and HCW support measures

HCWs were evaluated by the mental health care team (n = 165) during the post-duty isolation period by a semi-structured interview. Of these only, 10.9% (n = 18) were considered to have a clinically diagnosable anxiety disorder and/or mild depressive episode. Our HCW also reported insomnia (n = 23), isolation-related stress (n = 18), and fear of contracting COVID-19 infection (n = 32). On a Likert scale of 1–10, HCW scored stress as 5.7 ± 2.5 and fear of taking COVID-19 home as 6.5 ± 2.4. They also rated the accommodation provided as 7.8 ± 2.5 and PPE provided as 8.1 ± 2.8, with 88.9% reporting satisfaction with the COVID-19 care measures. About 869 (92.8%) of HCWs stated they were mentally prepared for the duty before entering the COVID-19 wing and 766 (81%) found the psycho-social support adequate during their isolation period.

## Discussion

Our large prospective study demonstrates that our HCW policy was effective in motivating frontline workers, PPE compliance, vaccine acceptance, and low infection rates during the first COVID-19 wave (0.23%) and during the second wave (23.7%). Our HCW protection model can be implemented in resource-poor settings and was successful even prior to vaccine availability, wherein PPE remains the mainstay of HCW protection, and therefore is replicable in future pandemics. An HCW-centric policy to support frontline workers complements the patient-care-centric model. In addition, the results of the vaccine serosurvey suggest a robust neutralizing antibody response in HCW suggesting sufficient humoral immunity. Overall immunization coverage was 94.3%, the level of seroprotection was 98.7 %. Although 224 of our HCW were PCR-positive confirmed infections, many had unreported subclinical infections suggesting, the majority had mild disease, with only 6 reporting moderate infection, and no deaths. Women HCW, and those with a family contact were at higher risk of COVID-19, there was no association with occupation, duration of duty, or site of deployment in the hospital risk zones.

Nguyen et al reported the prevalence of COVID-19 was 2747 cases per 100 000 frontline workers compared with 242 cases per 100 000 people in the general community. Compared with the general community, HCWs were at increased risk for COVID-19 test positivity (aHR 11·61, 95% CI 10·93–12·33).^16^ The strength of our unique HCW-centric care model is to ensure continuous rotation of trained HCW with holistic support to deal with future pandemics or new COVID-19 variants.^17^ (Figure 2)

Our conclusions and recommendations because of this study are as follows (i) PPE compliance and vaccine acceptance among our HCW is high (ii) Adequate PPE is central to preventing infection (iii) Post-vaccination serosurvey showed high levels of the neutralizing antibody. Protective antibody was higher in post-vaccine plus infection group vs only vaccination group. There were no cases of severe COVID-19 infection. SARS-ACoV antibody was reactive in 60.1% of persons of the 386 HCWs who underwent the serological testing suggesting significant subclinical or undiagnosed COVID-19 infection in our HCW. (iv) The need for virological surveillance to detect new variants of COVID-19 due to antigenic shifts or drifts is important, as this can prompt decisions for repeated boosters in HCW (v) Booster doses with heterologous vaccination or with new antigens incorporated in the existing vaccines need further assessment.^18,19^

In all cases of HCW infection, it is difficult to determine whether these were acquired in the hospital, but it is possible that many infections were community-acquired, as women, who are often caregivers at home were at increased risk of infection (and presence of a family member testing positive increased likelihood of HCW infection. Hence, HCWs should maintain precautionary measures in the community while adhering to the hospital PPE protocol.

A meta-analysis of 49 studies including 127,480 HCW showed overall seroprevalence of SARS-CoV-2 antibodies was 8.7% (95% CI 6.7%–10.9%) prior to vaccine availability. Male gender, Black Asian or Hispanic race, shortage of PPE, and household contact with suspected or confirmed cases of COVID-19 were associated with increased seropositivity.^20^ Among 2056 enrolled Canadian HCWs, 241 (11.7%) had positive SARS-CoV-2 serology, while 171 (71.0%) had prior COVID-19 infection.^21^ Another cohort showed seropositivity was associated with lack of physical distancing among HCWs in work areas and breakrooms.^22^ Periodic retraining can prevent aerosol-borne infections effectively, as indicated by our pre-vaccine era infection rate of 0.23%. New variants of SARS-CoV-2, influenza among others may be encountered and appropriate PPE usage and infection control measures remain the backbone of HCW protective measures.^23,24^

**Figure 3.**
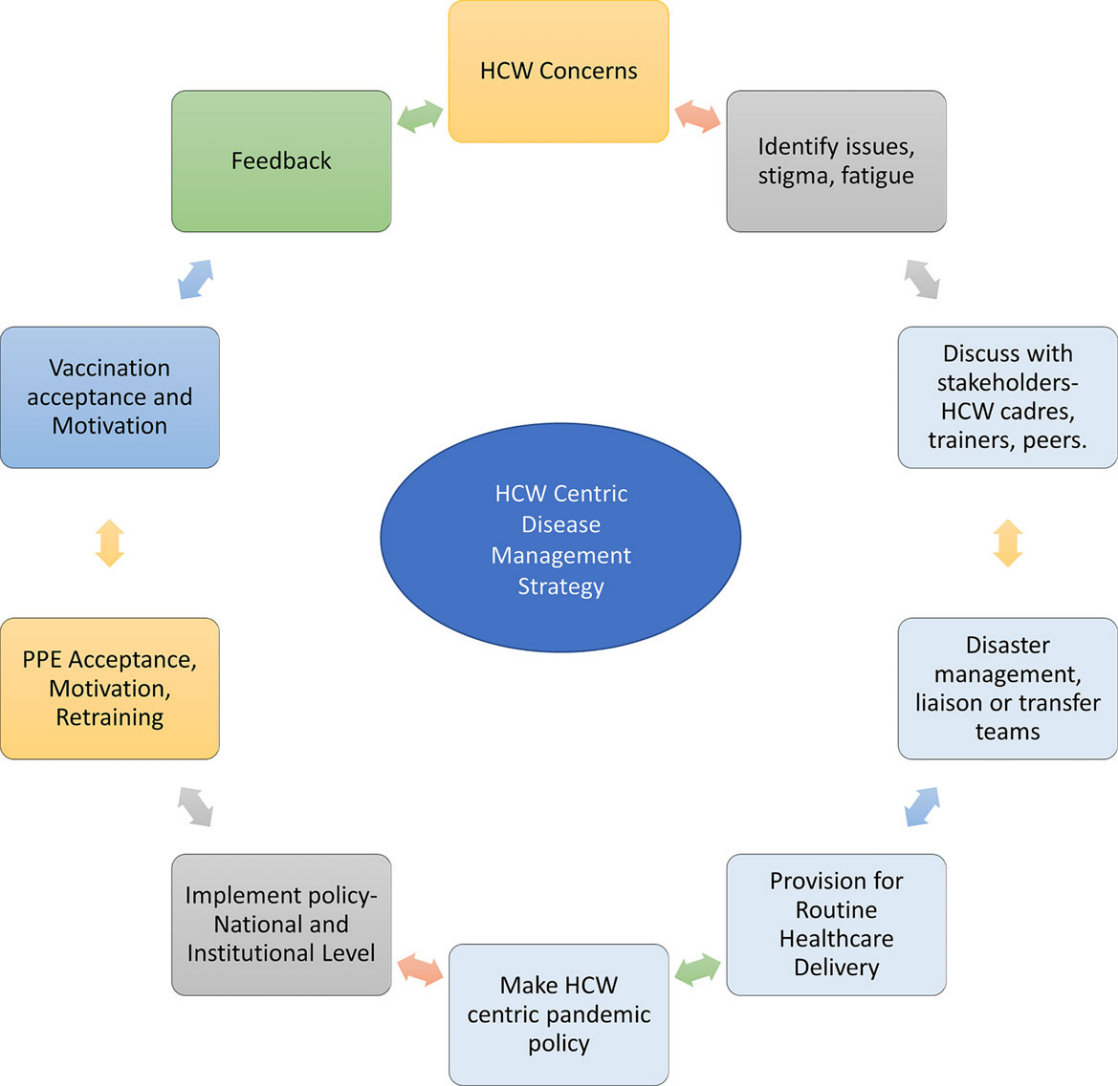
HCW-centric COVID-19 approach and Seroprotection status.

The strength of this study is the HCW-centric model of care, stratified serosurvey demonstrating adequate humoral immunity, and evidence-based advocacy for low-cost infection control measures like PPE provision and compliance, which are applicable to future pandemics.^25^ Although disease-centric management policies will change with new infections, an HCW-centric policy remains effective and reproducible. The creation of infectious disease wards, barrier patient-care areas, logistic measures like adequate PPE supply chains, safe accommodation, infection control training, and hospital planning will help prepare for the next pandemic. (Figure 3)

Our model effectively addresses the issue of COVID-19 burnout, while building a culture of systemic empowerment of HCW to deal with the sequelae of long COVID-19, while managing the deferred care for general health conditions.^26^ Motivation strategies like HCW training, assured provision of adequate PPE and rest, amelioration of social stigma, logistic support, mental healthcare access for depression, insomnia, post-traumatic stress disorder, and other complaints were central to our model.

Limitations of our cross-sectional serosurvey include the lack of data on cellular immunity and absence of fixed time points for testing for neutralizing antibodies after vaccination or infection. Whether the observed difference in antibody level translates to a difference in the duration of protection is not unclear, nor were we able to differentiate the antibody responses to different variants of concern.

## Conclusion

Our COVID care model was instrumental in protecting our HCW during the first and second waves, with low HCW infection rates. The provision of a precautionary dose of homologous vaccine may boost adaptive immune response in our HCW & reduce severity of infection but will not prevent HCW infections. Therefore, our study highlights that appropriate hospital planning, HCW training, and provision of appropriate PPE will be key to a robust sustainable health system response while protecting the healthcare workforce to address pandemic preparedness in the future

## Supporting information

Premkumar et al. supplementary material 1 Premkumar et al. supplementary material

Premkumar et al. supplementary material 2 Premkumar et al. supplementary material

## Data Availability

No additional data is available.

## Supplementary material

The supplementary material for this article can be found at https://doi.org/10.1017/ash.2023.459

## Patient and public involvement in research

This protocol deals with the creation of a novel hospital MDT and an HCW-centric policy whilst tackling the COVID-19 pandemic in our institution. Since this largely concerns HCW, and the process of creating a sustainable and willing healthcare team in a public health setting, the patient and public involvement is indirect as they were beneficiaries of this COVID-19 care model. The serosurvey was conducted in HCW who volunteered for testing.

## Cohort description

This manuscript deals with health policy and describes the management of human resources in the setting of COVID-19. Patients were not directly involved in the study and were managed as per standard of care at the institute.
