# Supplementary material for: Multipronged strategy for protection and motivation of healthcare workers during the COVID-19 pandemic: a real-life study
Source: Antimicrob Steward Healthc Epidemiol. 2024 Jul 25;4(1):e101. doi: 10.1017/ash.2023.459 (PMC11736454; doi:10.1017/ash.2023.459)
Supplement: Premkumar et al. supplementary material 1 — Premkumar et al. supplementary material [file S2732494X2300459Xsup001.docx]

HCW Supplement.

***Creation of the COVID-19 facility and Hospital Zones of COVID-19 Risk***

The Postgraduate Institute of Medical Education and Research (PGIMER), Chandigarh, India is a medical college/ university hospital and a nodal centre for COVID-19 care with 2800 beds catering to multidisciplinary referred cases from Northern India, specifically serving the states of Punjab, Haryana, Himachal Pradesh, and western Uttar Pradesh. We have 12600 full time employees across different cadres. Patients who tested positive were managed in the exclusive COVID-19 facility (red zone) with 323 beds, including 74 intensive care and 99 high-dependency beds.

All patients underwent risk assessment in the COVID-19 screening areas in the Emergency or severe acute respiratory illness (SARI) ward, and then moved to the appropriate hospital unit as per perceived risk of COVID-19. The area where patients were screened and where COVID suspects were retained for COVID testing until reports were made available was designated as **‘orange zone’** with medium risk of transmission. From the orange zone, on testing negative, they were moved to the general hospital services with low risk of transmission **(green zone)**, and if positive they were moved to a separate building of the hospital, which was designated as the COVID-19 facility **(red zone)**. This facility exclusively served confirmed COVID positive cases as it was a high-risk zone for transmission. The building had 5 levels, and each of which was designated for a separate function. The 1^st^ level was used for administration, and training of HCP, 2^nd^ ,3^rd^ and fourth levels were designated for stable patients requiring monitoring and the 5^th^ level was designated for patients requiring intensive care. Separate work areas, movement corridors and lifts were designated for the on-duty staff and administration to minimize risk to the health care workers (HCWs).

There was a multidisciplinary patient care unit created for expert COVID-19 patient care, as the main **COVID-19 patient centric health policy**. A separate HCW welfare unit was also created to protect and support their needs to ensure a willing rolling cohort of motivated HCW. This was part of the **HCW- centric health policy** to complement the patient care unit.

***Provision of HCW Protective equipment and Universal Precautions***

All HCW received training about use of personal protective equipment (PPE), HCW support measures (full board and transport), infection control measures, masking protocols, appropriate biohazard waste disposal and disinfection measures. The hospital PPE Committee, headed by Microbiology and Infection Control units, ensured PPE was provided as per the level of risk in the three zones. The Red and Orange zones were provided full PPE including N95 respirators, coverall suits, visor, cap, gloves, and dual shoe covers. In all areas with full PPE, the shift was limited to 6 hours, which avoided excessive workloads and allowed the HCW appropriate periods for rest and recreation. In case of any HCW becoming symptomatic or testing positive, isolation and contact tracing was done as per the Government of India advisories. The green zone HCW were provided triple layer surgical mask, surgical linen gowns for patient procedures and measures as per usual hospital procedures. [6] HCW training included pre- and post-duty protocol instructions, accommodation information, sample collection and safe transportation to the laboratory, communication skills and information about emergency contact helplines. Hands-on training for correct PPE ‘donning’ and ‘doffing’ was done by hands-on training.

As per a pan hospital advisory, universal infection control precautions were followed in all 3 zones including mandatory use of masks by HCW, patients and attendants, strict hand hygiene, thermal screening at entry, restriction of admissions based on triage priority, restriction of non-essential visitors to the hospital.

***Motivation Strategies for HCW***

We followed a multipronged approach to motivate our HCW. They received training pre-deployment to understand the duty requirements, and simplified workflows for patient care, sample collection and movement to ease their work-related anxiety. They were given adequate PPE and never faced any shortage or need for reuse. They were provided an escalation communication access to administrative, clinical, laboratory and HCW care unit personnel, to alleviate any task related problem or clinical emergency. The COVID-19 patient care unit had multidisciplinary faculty who had regular ward rounds and addressed patient care related issues. The HCW were made aware of the frequently asked questions by COVID-19 affected patients and families, and they were taught how to manage patients without compromising their own safety. Those who requested accommodation and transport were provided the facility for the duty period. The HCW had access to mental health professionals via video link or post duty interviews to ensure they were not under undue stress or could seek help when required.

Those who had completed their tour of duty were drafted into the training exercise to provide practical inputs and motivate the new recruits. We used the HCW’s smart phone to communicate with them as well as provide all required information/ services. The HCW were empowered to access COVID-19 related information, training modules via our web portal, communicate with the supervisory unit and coworkers, coordinate logistics and recreation using their personal mobile devices.

All these measures resulted in a low HCW infection rate during the first and second wave and ensured a rolling cohort of motivated personnel who were able to run the COVID-19 care facility while the rest of the team ensured routine non COVID-19 related patient care continued, albeit restricted.

When vaccines were available, out HCW were already motivated to be immunized. This is because Indian HCW are themselves involved in vaccination drives periodically, like the polio elimination drives, National Viral Hepatitis Elimination Programme, and childhood immunization campaigns. They were enthusiastic about taking the vaccine themselves and actively engaged with the community to ensure any misinformation was dispelled with positive reinforcement. This may contrast with western data wherein considerable vaccine reluctance was reported.

***Pre-deployment training of the HCP***

The eligibility criteria for front line workers in the red and orange zones were HCW aged <50 years, non-pregnant, non-lactating women, without apparent cause for immunosuppression, those without co-morbidities, and those who were willing to perform patient care duty in the COVID hospital as first responders. Many HCW volunteered in the first wave when there was widespread apprehension regarding COVID-19 in India, in HCW as well as the general community.

***HCW Support during the Duty Period***

Just before the duty, all HCW were provided with a high energy dry snack. The duty period was fixed at 6 hours so that restroom breaks and changes of PPE were minimized during the duty shift itself. Specified ‘donning’ rooms with mirrors with graphical step-by-step pictorial instructions were designed. Similarly, the ‘doffing’ areas had supervisors who could instruct the post duty HCW to remove the PPE layers correctly by direct instructions via CCTV. The duty area had a comfortable ambient temperature of 18-21^⸰^C, controlled with an area specific high-efficiency particulate air filtration handling unit. All HCW could communicate via their smartphone to report any breach in PPE, new symptoms, mental distress or need for any support. Lines of communication and escalation matrix for handling mental health, physical health and duty related issues was devised to ensure that the HCW were empowered and protected.

*Accommodation facility and transport*

A dedicated boarding and lodging facility was created for HCW who requested accommodation support during the duty and post-duty period if they were unable to isolate at home or feared community stigma. Many HCW continued to avail this facility even when the isolation requirement was removed as it provided peer support from coworkers and reduced their perceived fear of ‘carrying COVID-19 infection home’ especially in large households with limited space. All the HCW were periodically screened telephonically for any medical and mental health issues. Anxiety and depression were assessed using questionnaire (GAD-7 and PHQ-9), and if present they were counselled/ provided appropriate medication. Secure transportation facility was provided to HCW if they requested it.

**Results**

Supplementary Table 1: Exposure Risk and Vaccination details of our sero-surveyed HCW.

|  | **Total** | **Not Vaccinated** | | **Vaccinated** | |  |
| --- | --- | --- | --- | --- | --- | --- |
| **Parameter** | **All HCW** | **No COVID-19 Infection** | **Presumed COVID-19 Infection** | **No COVID-19 Infection** | **Presumed COVID-19 Infection** |  |
| **HCW number (%)** | **n=386 (100)** | **n=2 (0.5)** | **n=3 (0.8)** | **n=115 (29.8)** | **n=266 (68.9)** | **p value** |
| EXPOSURE DETAILS |  |  |  |  |  |  |
| Tours of Duty |  |  |  |  |  | 0.414 |
| 0 | 184 (47.7) | 1 (50.0) | 3 (100) | 53 (46.1) | 127 (47.7) |  |
| 1 | 77 (19.9) | 0 | 0 | 23 (20) | 54 (20.3) |  |
| 2 | 16 (4.8) | 1 (50.0) | 0 | 6 (5.2) | 9 (4.3) |  |
| 3 | 17 (4.4) | 0 | 0 | 3 (2.6) | 14 (5.3) |  |
| 4 | 17 (4.4) | 0 | 0 | 9 (7.8) | 8 (3.0) |  |
| 5 | 12 (3.1) | 0 | 0 | 5 (4.3) | 7 (2.6) |  |
| 6 | 3 (0.8) | 0 | 0 | 2 (1.7) | 1 (0.4) |  |
| 7 | 7 (1.8) | 0 | 0 | 2 (1.7) | 5 (1.9) |  |
| 8 | 53 (13.7) | 0 | 0 | 12 (10.4) | 41 (15.4) |  |
| Cumulative duration of Duty |  |  |  |  |  | 0.300 |
| Did not reply | 203 (52.6) | 1 (50.0) | 3 (100) | 50 (43.5) | 149 (56.0) |  |
| <1 week | 14 (3.6) | 0 | 0 | 4 (3.50) | 10 (3.8) |  |
| 1-4 wk | 39 (10.1) | 0 | 0 | 18 (15.7) | 21 (7.9) |  |
| 4-6 wk | 44 (21.6) | 1 (50.0) | 0 | 21 (18.3) | 22 (8.2) |  |
| 6-8 wk | 57 (14.8) | 0 | 0 | 13 (11.3) | 44 (16.5) |  |
| 2-4 months | 9 (2.3) | 0 | 0 | 2 (1.7) | 7 (2.6) |  |
| 4-6 months | 8 (2.1) | 0 | 0 | 3 (2.6) | 5 (1.9) |  |
| >6 months | 11 (2.8) | 0 | 0 | 4 (3.5) | 7 (2.6) |  |
| >1 year | 1 (0.3) | 0 | 0 | 0 | 1 (0.4) |  |
| VACCINATION DETAILS |  |  |  |  |  |  |
| **Covaxin^TM ®^** | 6 (1.6) | 0 | 0 | 3 (2.6) | 3 (1.1) |  |
| **Covishield^TM ®^** | 374 (96.9) | 0 | 0 | 112 (97.4) | 262 (98.5) |  |
| **Sputnik V ^TM ®^** | 1 (0.3) | 0 | 0 | 0 | 1 (0.4) |  |
| **None** | 5 (1.3) | 2 (100) | 3 (100) | 0 | 0 |  |
| Number of Doses |  |  |  |  |  |  |
| **Single** | 43 (11.1) | 0 | 0 | 11 (9.6) | 32 (12) | 0.000 |
| **Both doses** | 338 (87.6) | 0 | 0 | 104 (90.4) | 234 (88.0) |  |
| **None** | 5 (1.5) | 2 (100) | 3 (100) | 0 | 0 |  |
| Dose Interval |  |  |  |  |  | 0.001 |
| **Not vaccinated** | 5 (1.5) | 2 (100) | 3 (100) | 0 | 0 |  |
| **<4 wk** | 3 (0.8) | 0 | 0 | 2 (1.7) | 1 (0.4) |  |
| **4 wk** | 96 (24.8) | 0 | 0 | 34 (29.5) | 62 (16.06) |  |
| **4-6 wk** | 86 22.3) | 0 | 0 | 23 (20) | 63 (23.7) |  |
| **6-8 wk** | 45 (11.7) | 0 | 0 | 15 (13) | 30 (11.3) |  |
| **>8 wk** | 151 (39.1) | 0 | 0 | 41 (35.7) | 110 (41.4) |  |
|  |  |  |  |  |  |  |
|  |  |  |  |  |  |  |
|  |  |  |  |  |  |  |

**SARS-CoV 2 Serosurvey.**

SARS-CoV-2 specific neutralizing antibody responses characterised by IgG spike protein by chemiluminescence assay (CLIA) while total SARS-CoV-1 IgG was measured by Vitros Immunoassay (Orthoclinical Diagnostics, CEDEX, France). The Elecsys (Roche) Anti‑SARS‑CoV‑2 S assay uses a recombinant protein representing the receptor binding domain (RBD) of the Spike (s) antigen in a double-antigen sandwich assay format, which favours the quantitative determination of high affinity antibodies against SARS‑CoV‑2. The test is intended as an aid to assess the adaptive humoral immune response to the SARS‑CoV‑2 S protein. A result of ≥ 80U/mL is interpreted as positive for anti-SARS-CoV-2-S. The test was done on **Cobas e** 411 analyzer. Upon infection with SARS-CoV-2, the host usually mounts an immune response against the virus, typically including production of specific antibodies against viral antigens. IgM and IgG antibodies against SARS-CoV-2 appear to arise nearly simultaneously in blood.  Recent studies of antibody responses in patients with COVID-19 have associated higher titres of anti-N IgM and IgG at all time points following the onset of symptoms with a worse disease outcome. [2]

Moreover, higher titres of anti-S and anti-N IgG and IgM correlate with worse clinical readouts and older age, suggesting potentially detrimental effects of antibodies in some patients. [3]

There is significant inter-individual difference in the levels and chronological appearance of antibodies in COVID-19 patients, but median seroconversion has been observed at approximately two weeks. Age of the person can also affect antibody responses after infection or vaccination. [4]

Reister et al showed that a total of 1,610 samples from 402 symptomatic patients (including 297 samples from 243 hospitalized patients) with a PCR confirmed SARS-CoV-2 infection were tested with the Elecsys^®^ Anti-SARS-CoV-2 S assay. One or more sequential samples from these patients were collected at various time points after PCR confirmation. They showed 1,423 of the tested samples had a sampling date of 14 days or later after diagnosis with PCR. Of these 1423 samples, 1,406 tested as ≥0.8 U/mL in the Elecsys^®^ Anti‑SARS‑CoV‑2 S assay and hence considered positive, resulting in a sensitivity of 98.8 % (95 % CI: 98.1 – 99.3 %) in this sample cohort.[5]

High‑affinity antibodies can elicit neutralization by recognizing and binding specific viral epitopes Antibodies against SARS‑CoV‑2 with strong neutralizing capacity, especially potent if directed against the RBD, have been identified. The Elecsys anti-SARS-CoV-2 S assay exhibited its highest sensitivity (84.0%) at 15 to 30 days post-PCR positivity and exhibited no cross-reactivity, a specificity and PPV of 100%, and an NPV between 98.3% and 99.8% at ≥14 days post-PCR positivity, depending on the seroprevalence estimate.  [6] After infection or vaccination, the binding strength of antibodies to antigens increases over time - a process called affinity maturation. There are significant differences in the performance of SARS-CoV-2 antibody assays, especially with distance in time from PCR-confirmed COVID-19 infection. Allen et al compared clinical performance of these assays in 367 HCW with a confirmed PCR positive infection. Of those with previously confirmed infection, 41% (150/367) and 95% (348/367) tested positive on Abbott and Roche, respectively. At 21 weeks (150 days) after confirmed infection, positivity on Abbott started to decline. Roche positivity was retained for the entire study period (33 weeks). [7]

Overall, we found the SARS-CoV-2 antibody tests the best marker of detection of seroprotection in our HCW. This study was aimed at studying seroprotection status in Indian HCWs, not vaccine immunogenicity or time dependent kinetics of immune responses. Overall, the tests for neutralizing antibody are reasonable for use in a clinical setting.

**References**

1. Dutta U, Sachan A, Premkumar M, Gupta T, Sahoo S, Grover S, et al. Multidimensional dynamic healthcare personnel (HCW)-centric model from a low-income and middle-income country to support and protect COVID-19 warriors: a large prospective cohort study. *BMJ Open*. 2021;**11(2):**e043837. doi: 10.1136/bmjopen-2020-043837. PMID: 33619195; PMCID: PMC7902325
2. Tan W, et al. Viral kinetics and antibody responses in patients with COVID-19. *medRxiv.*2020 doi: 10.1101/2020.03.24.20042382
3. Jiang H-w, et al. Global profiling of SARS-CoV-2 specific IgG/IgM responses of convalescents using a proteome microarray. *medRxiv.*2020 doi: 10.1101/2020.03.20.20039495.
4. Yang HS, Costa V, Racine-Brzostek SE, Acker KP, Yee J, Chen Z, Karbaschi M, Zuk R, Rand S, Sukhu A, Klasse PJ, Cushing MM, Chadburn A, Zhao Z. Association of Age With SARS-CoV-2 Antibody Response. JAMA Netw Open. 2021 Mar 1;4(3):e214302. doi: 10.1001/jamanetworkopen.2021.4302. PMID: 33749770; PMCID: PMC7985726.
5. Riester E, Findeisen P, Hegel JK, Kabesch M, Ambrosch A, Rank CM, Pessl F, Laengin T, Niederhauser C. Performance evaluation of the Roche Elecsys Anti-SARS-CoV-2 S immunoassay. J Virol Methods. 2021 Nov;297:114271. doi: 10.1016/j.jviromet.2021.114271. Epub 2021 Aug 27. PMID: 34461153; PMCID: PMC8393518.
6. Higgins V, Fabros A, Kulasingam V. Quantitative Measurement of Anti-SARS-CoV-2 Antibodies: Analytical and Clinical Evaluation. J Clin Microbiol. 2021 Mar 19;59(4):e03149-20. doi: 10.1128/JCM.03149-20. PMID: 33483360; PMCID: PMC8092751.
7. Allen N, Brady M, Carrion Martin AI, Domegan L, Walsh C, Houlihan E, Kerr C, Doherty L, King J, Doheny M, Griffin D, Molloy M, Dunne J, Crowley V, Holmes P, Keogh E, Naughton S, Kelly M, O'Rourke F, Lynagh Y, Crowley B, de Gascun C, Holder P, Bergin C, Fleming C, Ni Riain U, Conlon N; PRECISE Study Steering Group. SARS-CoV-2 Antibody Testing in Health Care Workers: A Comparison of the Clinical Performance of Three Commercially Available Antibody Assays. Microbiol Spectr. 2021 Oct 31;9(2):e0039121. doi: 10.1128/Spectrum.00391-21. Epub 2021 Sep 29. PMID: 34585976; PMCID: PMC8557912.
