# Supplementary material for: Multipronged strategy for protection and motivation of healthcare workers during the COVID-19 pandemic: a real-life study
Source: Antimicrob Steward Healthc Epidemiol. 2024 Jul 25;4(1):e101. doi: 10.1017/ash.2023.459 (PMC11736454; doi:10.1017/ash.2023.459)
Supplement: Premkumar et al. supplementary material 2 — Premkumar et al. supplementary material [file S2732494X2300459Xsup002.docx]

HCW Supplement.

*Provision of HCW Protective equipment and Universal Precautions*

All HCW received training regarding use of PPE specific to their area, on infection control measures, mask protocols, waste disposal and disinfection protocols. PPE was provided as per the level of risk in the three zones. The red and orange zones had full PPE provision, with N95 respirators, coverall suits, visor, cap, gloves, and dual shoe covers. In all areas with full PPE, the shift was limited to 6 hours, which avoided excessive workloads, gave time for rest and recreation. In case of any HCW becoming symptomatic or testing positive, isolation and contact tracing was done as per the Government of India advisories. The green zone HCW were provided triple layer surgical mask, surgical linen gowns for patient procedures and measures as per usual hospital procedures. [6] Universal precautions were followed in all 3 zones including mandatory use of masks, strict hand hygiene, thermal screening at entry, restriction of admissions based on triage priority, restriction of non-essential visitors to the hospital and training of administration, security, sanitation, hostel and other personnel to reduce infections.

*Pre-deployment training of the HCP*

The eligibility criteria for front line workers in the red and orange zones were HCW aged <50 years, non-pregnant, non-lactating women, without apparent cause for immunosuppression, those without co-morbidities, and those who were willing to perform patient care duty in the COVID hospital as first responders. We prepared the HCW mentally for both the on-duty and post duty social isolation period by briefing them in a language understandable to them. Training included duty instructions, accommodation information, correct PPE protocols, sample collection and safe transportation to the laboratory, communication skills and information about emergency contact helplines. Hands-on training for ‘donning’ and ‘doffing’ of PPE was done by live demonstrations by faculty tutors. The remaining HCW were provided training in the form of online videos with instructional material from the infection control team.

*HCW Support during the Duty Period*

Just before the duty, all HCW were provided with a high energy dry snack as use of diapers was not acceptable to most. Specified ‘donning’ rooms with mirrors with graphical step-by-step pictorial instructions were designed. A few hand-signs were taught to all active workers for communicating via closed-circuit television (CCTV) cameras placed in the segregated donning and doffing room. The duty area had a comfortable ambient temperature of 18-21^⸰^C, controlled with an area specific high-efficiency particulate air filtration handling unit. Communication channels were established between the HCW and the COVID-19 Patient Care Unit using CCTV, computer, and smartphones. If anyone had a documented breach, they underwent a formal assessment to classify it as low or a high-risk exposure. Those with high-risk exposure, were counselled and quarantined in the private ward facility of our hospital. Those with low-risk exposure continued to work with self-monitoring and standard precautions.

*Accommodation facility and transport*

A dedicated boarding and lodging facility for HCW during the duty and post-duty period if they were unable to isolate at home or feared community stigma. Many HCW continued to avail this facility even when the isolation requirement was removed as it provided peer support from coworkers and allowed them to reduce potential risk of affecting their family, especially in large households with limited space. All the HCW were periodically screened telephonically for any medical and mental health issues. Anxiety and depression were assessed using questionnaire (GAD-7 and PHQ-9), and if present they were counselled/ provided appropriate medication. Secure transportation facility was provided to HCW if they requested it.

Results

Supplementary Table 1: Exposure Risk and Vaccination details of our sero-surveyed HCW.

|  | **Total** | **Not Vaccinated** | | **Vaccinated** | |  |
| --- | --- | --- | --- | --- | --- | --- |
| **Parameter** | **All HCW** | **No COVID-19 Infection** | **Presumed COVID-19 Infection** | **No COVID-19 Infection** | **Presumed COVID-19 Infection** |  |
| **HCW number (%)** | **n=386 (100)** | **n=2 (0.5)** | **n=3 (0.8)** | **n=115 (29.8)** | **n=266 (68.9)** | **p value** |
| EXPOSURE DETAILS |  |  |  |  |  |  |
| Tours of Duty |  |  |  |  |  | 0.414 |
| 0 | 184 (47.7) | 1 (50.0) | 3 (100) | 53 (46.1) | 127 (47.7) |  |
| 1 | 77 (19.9) | 0 | 0 | 23 (20) | 54 (20.3) |  |
| 2 | 16 (4.8) | 1 (50.0) | 0 | 6 (5.2) | 9 (4.3) |  |
| 3 | 17 (4.4) | 0 | 0 | 3 (2.6) | 14 (5.3) |  |
| 4 | 17 (4.4) | 0 | 0 | 9 (7.8) | 8 (3.0) |  |
| 5 | 12 (3.1) | 0 | 0 | 5 (4.3) | 7 (2.6) |  |
| 6 | 3 (0.8) | 0 | 0 | 2 (1.7) | 1 (0.4) |  |
| 7 | 7 (1.8) | 0 | 0 | 2 (1.7) | 5 (1.9) |  |
| 8 | 53 (13.7) | 0 | 0 | 12 (10.4) | 41 (15.4) |  |
| Cumulative duration of Duty |  |  |  |  |  | 0.300 |
| Did not reply | 203 (52.6) | 1 (50.0) | 3 (100) | 50 (43.5) | 149 (56.0) |  |
| <1 week | 14 (3.6) | 0 | 0 | 4 (3.50) | 10 (3.8) |  |
| 1-4 wk | 39 (10.1) | 0 | 0 | 18 (15.7) | 21 (7.9) |  |
| 4-6 wk | 44 (21.6) | 1 (50.0) | 0 | 21 (18.3) | 22 (8.2) |  |
| 6-8 wk | 57 (14.8) | 0 | 0 | 13 (11.3) | 44 (16.5) |  |
| 2-4 months | 9 (2.3) | 0 | 0 | 2 (1.7) | 7 (2.6) |  |
| 4-6 months | 8 (2.1) | 0 | 0 | 3 (2.6) | 5 (1.9) |  |
| >6 months | 11 (2.8) | 0 | 0 | 4 (3.5) | 7 (2.6) |  |
| >1 year | 1 (0.3) | 0 | 0 | 0 | 1 (0.4) |  |
| VACCINATION DETAILS |  |  |  |  |  |  |
| **Covaxin^TM ®^** | 6 (1.6) | 0 | 0 | 3 (2.6) | 3 (1.1) |  |
| **Covishield^TM ®^** | 374 (96.9) | 0 | 0 | 112 (97.4) | 262 (98.5) |  |
| **Sputnik V ^TM ®^** | 1 (0.3) | 0 | 0 | 0 | 1 (0.4) |  |
| **None** | 5 (1.3) | 2 (100) | 3 (100) | 0 | 0 |  |
| Number of Doses |  |  |  |  |  |  |
| **Single** | 43 (11.1) | 0 | 0 | 11 (9.6) | 32 (12) | 0.000 |
| **Both doses** | 338 (87.6) | 0 | 0 | 104 (90.4) | 234 (88.0) |  |
| **None** | 5 (1.5) | 2 (100) | 3 (100) | 0 | 0 |  |
| Dose Interval |  |  |  |  |  | 0.001 |
| **Not vaccinated** | 5 (1.5) | 2 (100) | 3 (100) | 0 | 0 |  |
| **<4 wk** | 3 (0.8) | 0 | 0 | 2 (1.7) | 1 (0.4) |  |
| **4 wk** | 96 (24.8) | 0 | 0 | 34 (29.5) | 62 (16.06) |  |
| **4-6 wk** | 86 22.3) | 0 | 0 | 23 (20) | 63 (23.7) |  |
| **6-8 wk** | 45 (11.7) | 0 | 0 | 15 (13) | 30 (11.3) |  |
| **>8 wk** | 151 (39.1) | 0 | 0 | 41 (35.7) | 110 (41.4) |  |
|  |  |  |  |  |  |  |
|  |  |  |  |  |  |  |
|  |  |  |  |  |  |  |
